# Supplementary material for: The impact of a randomized controlled trial of a lifestyle intervention on postpartum physical activity among at-risk hispanic women: Estudio PARTO
Source: PLoS One. 2020 Jul 24;15(7):e0236408. doi: 10.1371/journal.pone.0236408 (PMC7380594; doi:10.1371/journal.pone.0236408)
Supplement: S2 File — (DOCX) [file pone.0236408.s002.docx]

**ESTUDIO PARTO**

**PROYECTO PARA REDUCIR DIABETES TIPO DOS**

**PROJECT AIMING TO REDUCE TYPE TWO DIABETES**

**A RANDOMIZED TRIAL OF A POSTPARTUM DIABETES PREVENTION PROGRAM FOR HISPANIC WOMEN**

**PROTOCOL**

**Approach & Methods**

**D1. Study Overview**

**The overall goal of this randomized controlled trial is to test the efficacy of a culturally and linguistically modified, individually-tailored lifestyle intervention to reduce risk factors for type 2**

**diabetes and cardiovascular disease among postpartum Hispanic women with a history of abnormal**

**glucose tolerance during pregnancy.** Specific aims are to evaluate the impact of the intervention on 1)

*postpartum weight loss*, 2) biomarkers associated with insulin resistance (i.e., glucose, insulin, HbA1c, leptin,

TNF-α, HOMA, AUCgluc, adiponectin), 3) other cardiovascular risk factors (i.e., blood lipids, blood pressure,

CRP, fetuin-A, albumin-to-creatinine ratio), and 4) the adoption and maintenance of postpartum behaviors

associated with *weight loss* and prevention of diabetes risk (i.e., physical activity, diet). Eligible Hispanic

women will be recruited after routine GDM screening and randomly assigned to a Lifestyle Intervention (n=150) or a Comparison Health and Wellness (control) intervention (n=150). The intervention will be based on our efficacious exercise and dietary interventions for Hispanics (R01NR011295; *WIC Common Pathways*).

Multimodal contacts (i.e., in-person, telephone counseling, and mailed print-based materials) will be used to

deliver the intervention from randomization *(29 wks gestation)* through delivery, and for 12 months postpartum. Targets of the intervention are to achieve *Institute of Medicine Guidelines for postpartum weight loss*; American College of Obstetrician and Gynecologist guidelines for physical activity; and American Diabetes Association guidelines for diet. The intervention draws from Social Cognitive Theory and the Transtheoretical Model and addresses the specific social, cultural, economic, and physical environmental challenges faced by underserved Hispanic women. Measures of adherence will include accelerometers and dietary recalls. The project is a Renewal Application of R01 DK064902, a study of lifestyle risk factors for GDM in Hispanic women. The proposed project builds upon the expertise of the investigative team in conducting randomized controlled trials of lifestyle interventions among Hispanic pregnant women (R01 DK074876; *S3948 ASPH/CDC*) and controlled trials of lifestyle interventions among low-income Hispanics with type 2 diabetes and pre-diabetes (R18 DK0658850; R18DK067549) and can readily be translated into clinical practice in underserved and minority populations.

**D2. Study Population & Eligibility Criteria**

The study will be based at the ambulatory obstetrical practice of Baystate Medical Center in Western Massachusetts. Baystate Medical Center is the site of the parent grant and is a large tertiary care facility; 4,300 infants are delivered annually and 57% are Hispanic (predominantly Puerto Rican). The study site practices universal screening for GDM, which consists of a random 50- g glucose load and a plasma glucose determination 1 hour later. If the plasma glucose value is >135 mg/dL, a diagnostic 100-g 3 hour OGTT is conducted. Eligible women will be Hispanic women with one or more glucose values during the screening or diagnostic test meeting or exceeding the thresholds defined according to the American Diabetes Association.^103^We will exclude women with: 1) history of type 1 or type 2 diabetes, heart disease, high blood pressure, or chronic renal disease, 2) contraindications to postpartum participation in moderate physical activity or a low-fat/high-fiber diet (e.g., Crohn’s disease, ulcerative colitis), 3) inability to read English or Spanish at a 6th grade level, or 4) <16 or >45 yrs of age. Women who have a preterm birth after enrollment or a stillbirth will not be excluded as the focus is on maternal postpartum risk for subsequent chronic disease. Women carrying multiples will be excluded, as will women who are greater than 37 weeks gestational age.

**D3. Recruitment, Baseline Assessment, and Sample Size**

Prenatal care patients will be recruited by a bilingual (Spanish and English) health educator following one or more glucose values meeting or exceeding the thresholds defined according to the American Diabetes Association (~24-28 wks gestation). Women will be informed of the aims and procedures of the project, sign an informed consent, and complete a brief screening form to determine eligibility. The baseline assessment will be conducted (see Figure 1) . Each assessment and blood draw and each returned tailoring questionnaire is associated with a gift card for a total of up to $270 to be handed out after completing the study component.

Based on recruitment rates observed in our prior studies (R01DK074876, and R01 DK064902), we conservatively expect to recruit a total of 300 women over the course of the study (150 in each treatment arm) (see Timeline D.19). We conservatively estimate a 15% rate of loss to follow-up. This would result in a final sample size of 128 participants in each treatment arm upon which our power calculations are based

Patients will be recruited after the time of their routine gestational diabetes screening. Patients with an abnormal value on the 1 hour glucose tolerance test will be approached at their 3 hour glucose tolerance test, will be mailed a letter and/or called with information on the study. Patients may also be referred from their routine meeting with a Baystate nutritionist after having an abnormal glucose value on the 1 and/or 3 hour test.

The recruitment sites will include the Wesson Women’s Clinic, Baystate Midwifery and Women’s Health, Baystate Maternal-Fetal Medicine and the Baystate Perinatal Diagnostic Center. Screening is done prior to the patient’s visit by a research assistant. A Centricity appointment report is reviewed which provides all women with OB appointments in the above locations. This report has the patients’ name, medical record number, date of birth, ethnicity, last menstrual period, appointment reason, appointment time and location. This information will be combined with lab results from all 1 hour glucose tolerance tests performed at Baystate Medical Center.

In addition, a list of women with abnormal glucose levels based on their routine 1-hr glucose tolerance test will be created by the Baystate Reference Lab and given to Peter St. Marie once per week for staff members to review.

Based on information from the Baystate nutritionist, the Centricity roster and the list of women with abnormal glucose levels from the Baystate Reference Lab, the research assistants will develop a potential participant list. This list will be maintained as a project on the UMASSMED REDCap server. This project will only be accessible to Baystate IRB approved staff. The pre-screened patients are then approached to determine if they are interested in participating in the study. If they are not eligible, the research assistant enters their status and/or reason for ineligibility. If women are eligible and interested, they will be informed of the aims and procedures of the project, sign an informed consent and HIPAA release, and the research assistant will initiate study procedures. If this cannot be completed in person, it will be completed over the telephone.

One purpose of the potential participant list is to create a "Do Not Approach" report which recruiters would be able to review prior to approaching subjects. Prenatal patients have frequent visits and there are several recruiters for this study. A REDCap report will be produced which will be compared with the daily appointment report, so that recruiters can avoid re-approaching subjects who have already been recruited, have refused or have been determined to be ineligible, thereby limiting potential participant burden. In addition, at the conclusion of the study the data from this the potential participant list will be used to provide counts of subjects approached but found ineligible and their reason for ineligibility. The purpose for this data is to better describe the non-participants which will help determine any potential selection bias in this study. After the de-identified counts are tallied, the results will be sent to UMASS and the potential participant list will be destroyed. Only the Baystate PI and research coordinator will have the user rights to export data from the REDCap project. Identified data from this list will never be sent outside of Baystate.

**
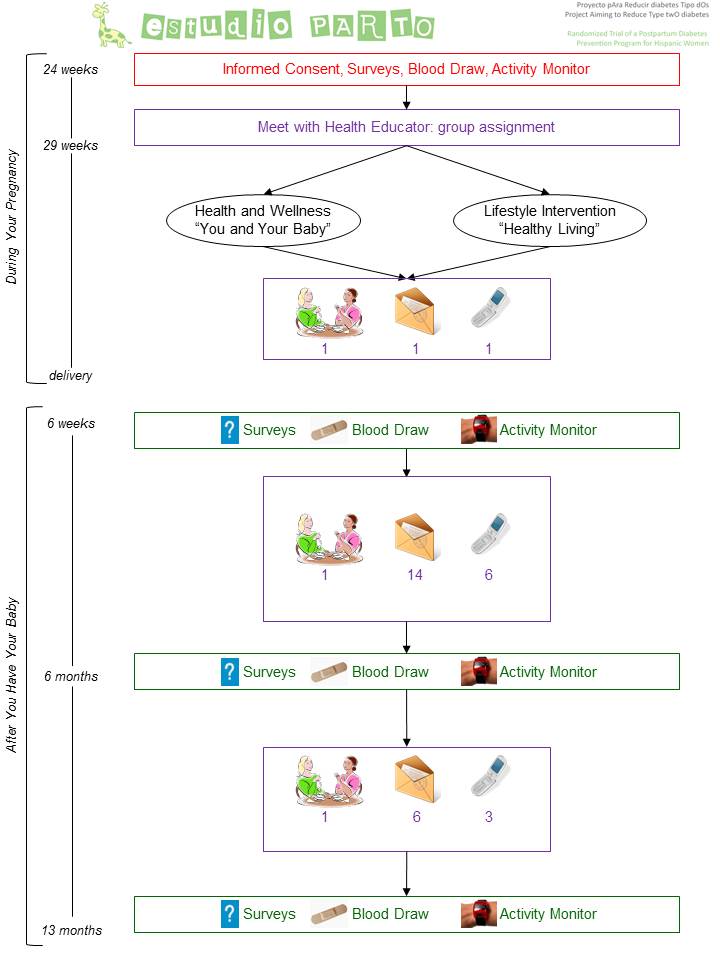
Figure 1 : Study Design and Data Collection Time Points**

**D.4. Retention**

Our team has a strong track record of retaining participants. Central to our retention strategy are bicultural/bilingual health educators that can build rapport with participants as well as materials that are culturally congruent (developed based on focus groups among Hispanic women, 3R01DK074876, R21NR009864, DK0658850). To further ensure retention, prior to randomization, the health educators clearly delineate the requirements of the study. Continued contact with participants over the course of the intervention (e.g., telephone calls, mailings, and assessments) also improves retention. In addition, the proposed study design facilitates high retention:

1) Enrollment occurs prior to delivery, a time period during which we have a track record of success in recruitment (Sections c.1 and c.2).

2) The kickoff face-to-face meeting will take place at the time of routine 6 wk postpartum visit. Adherence to this visit is facilitated by free bus passes, and the 2006 Mass. insurance reform law providing free health care insurance for residents earning <150% of the federal poverty level. However, if a woman does not intend to attend this visit, the health educator will meet the women at locations outside the hospital, with consideration for the participant’s and the health educator or assessors level of comfort (i.e., their pediatrician’s office, their home, a community center). Locations will be selected for privacy (for example, an unused exam room at a pediatrician’s office) and only staff who have experience making home visits will be permitted to complete home visits.

3) The 6- and 12-mos assessments will be conducted at Baystate, or the participants will be met at locations outside the hospital, with consideration for the participant’s and the health educator or assessors level of comfort (i.e., their pediatrician’s office, their home, a community center). Locations will be selected for privacy (for example, an unused exam room at a pediatrician’s office) and only staff who have experience making home visits will be permitted to complete home visits.

4) Delivering the intervention does not rely upon women remaining in the immediate area during the postpartum year as it is based upon tested individually targeted mailed print-based materials and telephone contacts. In addition to providing home/cell/work telephone numbers, women will be asked to provide the contact information for a person close to them.

**D.5. Randomization**

Prior to randomization (~29 wks gestation) all participants will be evaluated for study inclusion by asking if they have had their physical activity restricted by their prenatal care provider. If they answer yes, their medical records may be reviewed and or their provider may be contacted by Dr. Markenson (Co-Investigator, and Director of Maternal/Fetal Medicine at Baystate). Dr. Markenson may review the medical record or contact the subject’s provider of all enrolled participants if there is any concern about the safety for the subject to participate in the study, but “clearance” from Dr. Markenson is only required for participants who answer yes to the restricted physical activity question . Eligible patients will be randomized into either the Lifestyle Intervention vs. the Health and Wellness Comparison Group. Randomization will be stratified based on study site and the results of the diagnostic 3 hour oral glucose tolerance test: 1) pass the diagnostic test; or 2) at least one glucose value during the diagnostic test meeting or exceeding the ADA thresholds. Within each strata, a blocked randomization will be used such that both treatment groups will be assigned an equal number of times in each set of 4 sequentially enrolled patients. Participants enrolled into the Lifestyle Intervention will again be evaluated after delivery by asking if they have had their physical activity restricted at their postpartum visit with their provider. Participants who answer yes will not proceed further in the study until Dr. Markenson has either reviewed their medical record or contacted their provider and cleared them for further participation. Dr. Markenson reserves the right to review the medical record of all enrolled participants, but “clearance” from Dr. Markenson is only required for participants who answer yes.

**D.6. Intervention**

The Lifestyle Intervention is an evidence-based approach utilizing culturally modified, motivationally targeted, individually-tailored intervention materials from our randomized controlled trials for overweight pregnant Hispanics (ASPH/CDC S3948 PI: Chasan-Taber, Co-I Rosal, Marcus, Stanek), for pregnant women at high risk of GDM (R01DK074876, PI: Chasan-Taber, Co-I Marcus, Braun, Markenson, Tucker), for postpartum weight loss among overweight and obese low-income women enrolled in WIC (Common Pathways, PI: Rosal), as well as our randomized controlled trials testing the mailed print-based materials among Hispanic women (R01NR011295, R21NR009864, R01HL64342, PI: Marcus). These programs capitalize on efficacious behavioral weight loss strategies. The intervention begins in late pregnancy (~29 weeks gestation) and continues through the first year postpartum. It consists of 3 in-person sessions, 10 phone calls and 21 mailings (see Figure 1).

Overall Goals: are to achieve the IOM Guidelines for postpartum weight loss and to improve maternal metabolic status by achieving and maintaining 1) postpartum weight reduction to prepregnancy weight if prepregnancy BMI was in the normal range, or achieve a 5% reduction from prepregnancy weight if prepregnancy BMI was overweight/obese, as recommended by the IOM;^114^ 2) at least 150 min per week of moderate intensity physical activity such as brisk walking, as recommended by ACOG,^115^ and 3) reduction in postpartum total caloric intake via reduced consumption of popular calorie dense foods (e.g., fast food, high-fat snacks, fried foods and sugar-sweetened beverages), reduced portion size, appropriate modifications in ethnic recipes, and higher fruit and vegetable intake, as recommended by the American Diabetes Association.^116^

**D.7. Theoretical Framework**

The intervention draws from Social Cognitive Theory and the Transtheoretical Model and takes into account findings by our research group on the specific social, cultural, economic, and environmental resources as well as challenges faced by women of Hispanic backgrounds.

The Transtheoretical Model was originally developed by Prochaska et al. to help understand how and when individuals make behavior change.^111^ According to this model, individuals adopting a new behavior move through a series of stages: Precontemplation (not intending to make changes), Contemplation (considering a change), Preparation (making small changes), Action (actively engaging in the behavior) and Maintenance (sustaining the change over time). Also included in this model are processes of change which constitute strategies that individuals use to help make changes in their behavior.

Theory-driven interventions using the Stages of Motivational Readiness for Change Model and Social Cognitive Theory have been shown to be effective for exercise adoption and dietary change.^13^ Interventions matched to the individual’s stage of motivational readiness for physical activity adoption have been recommended and utilized^106, 107^ and have been found to be more effective than non-targeted interventions.^15^

Using Social Cognitive Theory, the intervention will target patients’ perceptions/beliefs as well as their behavioral patterns. Central to Social Cognitive Theory is the construct of self-efficacy, an individual’s belief in his/her ability to perform a specific behavior in a specific situation (e.g., selecting low-fat food for lunch during a stressful day). Social Cognitive Theory posits that individuals develop self-efficacy for specific behaviors through their own ability to perform the behavior in a given situation (mastery experiences), watching others perform the behavior in specific situations (modeling), and by verbal persuasion from people in their environment that are able to perform the behavior.

Our intervention will provide a variety of opportunities for mastery experiences, modeling and verbal persuasion to enhance self-efficacy for self-management.

**D.8. Intervention: Introductory Phase** (about 29 weeks gestation to delivery) (Figure 1)

The intervention will start with a face-to-face session building upon the usual GDM management received by patients during this time period. Specifically, the session will target knowledge and attitudes regarding postpartum weight loss and type 2 diabetes prevention, with the goal of moving women over the continuum of pre-contemplation to contemplation and preparation for the Postpartum Active Intervention Phase. Women will be advised in accordance with the IOM guidelines for gestational weight gain, ACOG guidelines for pregnancy physical activity, and ADA guidelines for diet. This session will be followed over the remainder of pregnancy (Figure 1) by culturally modified, individually-tailored, motivationally targeted Mailed Print-Based Intervention Materials and Booster Telephone Counseling Sessions which will address gestational weight gain management and reinforce preparation for postpartum weight loss.

**D.9. Intervention: Active Phase** (~6 wks postpartum – ~6 mos postpartum) **and Maintenance Phase** (~6 – 13 mos postpartum) (Figure 1)

The active phase will start with a face-to-face session. The session will include administration of questionnaires that will facilitate tailoring the intervention: an Exercise Tailoring Questionnaire, and a dietary tailoring questionnaire.^117^ Counseling will include the development of individualized weight loss, physical activity, and dietary goals as described below. This session will be followed by culturally modified, individually-tailored, motivationally targeted Mailed Print-Based Intervention Materials based on responses to monthly mailed Tailoring Questionnaires and Booster Telephone Counseling Sessions. Each mailed component is in Spanish and English and is written at a 6th grade reading level. Quality control procedures ensure that stage of change and social cognitive constructs are consistently represented in all intervention materials. Systems created in our pilot study, “Estudio Vida,” ensure that all mailings (i.e., physical activity and dietary intervention materials) are synchronized such that participants receive them at the same time. Telephone sessions facilitate review of progress toward the weight loss goal and provide 1) motivationally-based individualized feedback (i.e., problem-solving of challenges such as balancing caregiver/household responsibilities, cultural norms of self-sacrifice, social support, partner negotiation, and neighborhood safety), 2) review of weight loss grids and activity and dietary logs to evaluate progress toward behavioral goals, and 3) new goal setting. Telephone Sessions and Mailed Print-Based Materials continue into the **Intervention: Maintenance Phase** (~6 – 13 mos postpartum) on a bimonthly schedule.

**D.10. Curriculum**

At the outset of the intervention, a weight-loss goal based on prepregnancy BMI will be set and participants will be encouraged to work toward this goal by focusing on a reduction of 1-2 lbs/wk. The health educator will utilize a checklist of motivators for wanting to lose weight specific to new mothers to highlight benefits of weight loss and reinforce engagement. Participants will be provided with a digital scale and be encouraged to weigh themselves at home daily and chart their weight on a grid weekly. Emphasis will be placed on using the weight grid to better regulate diet and exercise behaviors. Mailed Stage Matched Manuals and Tip Sheets include strategies to elicit family and social support for weight loss and tips for managing fluctuations in weight loss motivation over time.

Physical Activity Change will be targeted via individualized week-by-week physical activity goals which focus on increasing, by 10%, the time spent in moderate activity as well as steps. Women will choose what form of safe activity they enjoy the most or can most readily fit into their lifestyle, from dancing to walking in a shopping mall to yard work. The accumulation of short bouts (i.e., 10 min episodes) will be encouraged. Mailed Stage-Matched Manuals matched to the participants’ current level of motivational readiness for change focus on the benefits of exercise, building social support for new behavioral patterns, and strategies for overcoming barriers to exercise specific to Hispanic women. Research staff at University of California, San Diego (UCSD) will analyze coded responses from participants’ tailoring questionnaires using states of change analysis from the transtheoretical model. They will then provide UMass Amherst with the tailored intervention that is most appropriate for the participant. Based on responses to the tailoring questionnaires, individually-tailored computer Expert-System Feedback Reports^85-88^ draw particular messages from a library of approximately 296 messages regarding motivation, self-efficacy, and cognitive and behavioral strategies for exercise adoption. Mailed Tip Sheets include topics such as stretching and exercising with baby

(e.g., walking while pushing a stroller). Pedometers (Omron) and activity logs are provided for women to monitor their activity. Prior studies support the use of pedometers as a motivational tool for walking.^118^

Dietary Change will be targeted via individualized week-by-week dietary goals determined in conjunction with the participant from an individualized dietary target goals checklist and based upon the woman’s caloric intake, breastfeeding status, and stage of change for a variety of dietary changes. Calories are targeted through the identification of high-calorie, high-frequency food items in the woman’s diet. Participants are provided with a Food Calorie Guide for culturally relevant foods (Appendix II). Actionable behavioral substitutions will emphasize what to eat and what not to eat, and steps to trying healthier methods for preparing traditional ethnic recipes. Mailed Tip Sheets cover the range of eating triggers in this population and strategies to manage these challenges, tips on meal planning, healthy recipes and grocery shopping lists, food label reading instructions with attention to cultural preferences. Participants will be provided with measuring cups and instruction in tracking caloric intake on a dietary log.

**D.11. Comparison Health & Wellness Intervention**

To ensure retention and to control for contact time, the Comparison Health & Wellness arm will receive mailed health materials and telephone booster calls on the same schedule as the Lifestyle Intervention arm. In this way, we control for contact time, while keeping the content of the two interventions distinct. These booklets represent high-quality, standard, low-cost, self-help material currently available to the public and focus on non-exercise and non-dietary topics in both English and Spanish. Hispanic control-arm participants in our prior studies (R01DK074876, R01NR011295, R21NR009864) reported that these materials were of interest^128^ and differential dropout did not occur between study arms.

**D.12. Outcome Variables**

**D.12.1. Biomarkers of Insulin Resistance and Other CVD Risk Factors**

Fasting postnatal samples will be collected at a Baystate Reference Laboratory before 30 weeks gestation, 6 weeks postpartum and 6 and 13 months postpartum (termed “blood draw”). Samples will be stored at -80 degrees Celsius in a freezer at Baystate with temperature monitoring, alarm and a back-up power system. Samples will be shipped periodically to the assaying lab. Residual blood samples will be stored at Dr. Rafai’s lab only to resolve potential data validation questions related to this study, and will not be used for any other study. The residual samples will be destroyed 7 years after study completion (December 2021). Quality control for specimen handling will be performed monthly by the head lab technician. Samples will be assayed by Dr. Nader Rifai (Department of Laboratory Medicine, Harvard University) at Children’s Hospital, Boston, MA. **Fasting Glucose** (FG) will be measured enzymatically on the Roche P Modular system using Roche Diagnostics reagents (Indianapolis, IN).^135^ Glucose at the concentrations of 90 and 312 mg/dL are determined in Dr. Rifai’s laboratory with a day-to-day variability of 1.7 and 1.6%, respectively. **Fasting Insulin** (FI) will be measured by an electrochemiluminescence immunoassay on the Roche E Modular system. The lowest detection limit of this assay is 0.2 uU/mL and the day-to-day imprecision values at concentrations of 6.36, 20.9 and 747 uU/mL are 2.6, 2.8 and 2.5%, respectively. The **Hemoglobin A1c (HbA1c)** determination on the Roche P Modular system will be based on turbidimetric immunoinhibition using packed red cells. The day-today variability at values of 5.5 and 9.1 are 1.9 and 3.0%, respectively. Total **Adiponectin (Multimeric)** will be measured using an ELISA method from ALPCO Diagnostics Inc. (Salem, NH). The day-to-day variabilities at 9130 and 3930 are 9.8, 10.2%, respectively. **Leptin** will be measured by an ultra-sensitive ELISA assay, an enzymatically amplified "two-step" sandwich-type immunoassay (R&D Systems, Minneapolis, MN). The day-to-day variability at concentrations of 65.7, ^146^ and 581 pg/mL are 5.4, 4.2 and 3.5%, respectively. **Lipoprotein Profile:** will be simultaneously performed on the Roche P Modular system. Dr. Rifai’s laboratory is certified by the CDC/NHLBI Lipid Standardization Program.^136^ **Total** **Cholesterol** will be measured enzymatically.^137^ At cholesterol concentrations of 132.8 and 280.4 mg/dL, the day-to-day reproducibility are 1.7% (SD=2.4 mg/dL) and 1.6%, respectively. **Triglycerides** will be measured enzymatically with correction for endogenous glycerol.^138^ Triglycerides at concentrations of 84.0 and 201.8 mg/dL have a day-to-day reproducibility of 1.8% (SD=1.6) and 1.7% (SD=3.5), respectively. The concentration of **High** **Density Lipoprotein Cholesterol** will be determined using a direct enzymatic colorimetric assay.139 HDL-C at the concentrations of 27.0 and 54.9 mg/dL have a day-to-day reproducibility of 3.3 (SD=0.9) and 1.7% (SD=0.9), respectively. **Low Density Lipoprotein Cholesterol** will be determined by a homogenous direct method.^140^ The day-to-day variabilities at concentrations of 90, 106, and 129 mg/dL are 3.01, 2.34 and 2.18%, respectively. The concentration of **High Sensitivity C-Reactive Protein (hsCRP)** will be determined using an immunoturbidimetric assay on the Roche P Modular system using reagents and calibrators from DiaSorin (Stillwater, MN). The day-to-day variabilities of the assay at concentrations of 0.91, 3.07 and 13.38 mg/L are 2.81, 1.61 and 1.1%, respectively. **TNF-**a**-receptor II** is measured by an ELISA assay from R&D Systems. The assay employs the quantitative sandwich enzyme immunoassay technique. The day-to-day variabilities of the assay at concentrations of 89.9, 197, and 444 pg/mL are 5.1, 3.5, and 3.6%, respectively. **HOMA:** Homeostasis model assessment will be calculated as [(FI x FG)/22.5] on a scale of 1-8. **Area-under-the-glucose curve (AUCgluc)** will be integrated over time using the trapezoidal rule.^141^ **Fetuin-A** will be measured by an enzyme immunoassay (EIA) (BioVendor – Candler, NC). The procedure follows the basic principle of enzyme immunoassay where there is competition between an unlabeled antigen and an enzymelabeled antigen for a fixed number of antibody binding sites. The assay possesses a sensitivity of 0.35 ng/mL and a run-to-run imprecision at Fetuin-A concentrations of 12.8 and 27.2 ng/mL of 5.1 and 2.6%, respectively. **Albumin-to-creatinine ratio (ACR)**: Albumin will be measured by a colorimetric assay, an automated dyebinding method using the Roche P Modular system and Roche Diagnostics reagents (Indianapolis, IN). The day-to-day variabilities at concentrations of 2.48 and 3.87 g/dL are 2.75 and 1.91%, respectively. Creatinine will be measured by an enzymatic method using the Roche P Modular system and Roche Diagnostics reagents (Indianapolis, IN). Creatinine at the concentrations of 1.54, 1.61 and 3.69 mg/dL have a day-to-day variability of 2.1, 1.6 and 2.5%, respectively. **Postpartum Diabetes Screening** will occur at each assessment and will follow the guidelines of the 2007 5th International Workshop Conference on GDM recommending a postpartum 75-g OGTT ^142^ using diagnostic criteria defined by the American Diabetes Association.^109^

**D.12.2. Covariates**

**Clinical Characteristics of the Current Pregnancy:** will be abstracted from the pregnancy medical record and include study site. Weight is measured prospectively at each prenatal visit and postpartum weight will be measured during home visits by trained study staff to the nearest 0.1 kg on accurately calibrated standard clinical scales using a standardized protocol. Gestational age will be based upon ultrasound assessment. Gestational weight gain will be calculated as the difference between maternal weight at delivery and prepregnancy weight. Adherence with IOM weight gain guidelines will be calculated by comparing the observed weight gain with the 2009 IOM Guidelines. 114 We will also collect gestational age at the time of GDM screen; degree of abnormality on glucose tolerance testing during pregnancy (i.e., the randomization stratification criteria); treatment for abnormal glucose tolerance during pregnancy (e.g., diet, oral hypoglycemic and/or insulin); pregnancy complications (e.g., hypertensive disorders of pregnancy, infection); birth outcomes

(e.g., birth weight, gestational age). Fasting glucose and insulin and HbA1c will be obtained at the time of the GDM screen along with a full lipid panel. Levels of these biomarkers differ during pregnancy vs. postpartum,^143^ and therefore they do not reflect baseline measures, but instead will be used as adjustment factors in regression models. **Medical History:** will include: personal history of GDM, family history of diabetes, previous infant with anomalies, still birth, or macrosomia, infertility, and parity. **Sociodemographic Factors**: will be collected via self-report at enrollment and include income, level of education, and health insurance; acculturation via the Psychological Acculturation Scale,^144^ language preference, and generation in the U.S.; Hispanic subgroup. **Smoking and Substance Use:** Smoking will also be assessed in Dr. Rifai’s lab via the biomarker cotinine, the major metabolite of nicotine (API – 3000, Applied Biosystems, CA). The assay is sensitive to 0.6 ng/mL; such that the assay can not only distinguish smokers from non-smokers (>10 ng/mL), but also cotinine levels obtained by passive exposure to smokers. Run to run precision at cotinine levels of 33,

124 and 268 ng/mL are 7.2, 5.6 and 3.6%, respectively. Alcohol consumption and drug use will be collected via self-report at each assessment period using questions from the Pregnancy Risk Assessment Monitoring

System.^145^ **Postpartum Factors:** will be collected via self-report at each postpartum assessment and include sleep via The Pittsburgh Sleep Quality Index (PSQI),^146^; depression via the Edinburgh Postpartum Depression

Scale^147^ validated in Hispanics.^148^ **Breastfeeding:** History of breastfeeding and frequency and duration of current breastfeeding (i.e., exclusive breast feeding, percentage of mixed breast and formula feeding, exclusive formula feeding), timing of introduction of solids, and other breastfeeding behaviors and beliefs will be assessed via a modified version of the validated Infant Feeding Questionnaire.^2^

**D.12.3. Measures of Adherence**

Measure of Adherence with Exercise: Women will wear the Actigraph activity monitor (Actigraph LLC, Pensacola, FL) on the wrist for a 4 day period at each of the 4 assessments. The GT3X-plus actigraph is currently being used to measure activity among pregnant women in NHANES and provides activity counts, steps, MET-min/week, time in sedentary behavior, patterns of active and sedentary behavior, % wear time, subject position, and periods when the device has been removed. Pregnancy cutpoints will be based on our prior studies.^63, 126, 77^ The GT3X plus can be worn during periods of sleep to measure the amount and quality of sleep. Previous studies have reported reasonable validity under laboratory conditions among pregnant women^158^ as well as under free living conditions.^124^Measure of Adherence with Exercise (Questionnaire Measure): Assessors, blinded to study arm, will administer the Pregnancy Physical Activity Questionnaire (PPAQ) developed and validated by our research team in the study population (NIH-NICHD R03-39341, PI: Chasan-Taber)^126^ and recommended for use in pregnancy and postpartum. Activity-specific measures will be summed to arrive at total daily energy expenditure (MET-hrs/wk) and further classified into categories based on activity type (i.e., sports/exercise, household/caregiving, occupational, transportation) and intensity (i.e., light, moderate, and vigorous).

Measure of Adherence with Diet (24-Hour Diet Recalls): Assessors, blinded to study arm, will conduct 3 24-hour diet recalls during each assessment period.

**D.13. Measures of Acceptability/Feasibility**

After study completion, the assessor will administer a Satisfaction Survey which will assess a variety of factors relevant to the acceptability and feasibility of the intervention. For example, women will be asked what attracted them to the study, what barriers/facilitators enabled them to continue to participate, their response to the intervention materials, their perspectives on the assessment materials as well as the number of measures. Women who drop out of the study will also be contacted for a similar discussion which will include asking women what we could have done to keep them more engaged and barriers to participation. At the end of the study, staff feedback will also be queried using structured satisfaction surveys.

**D.14. Staff Training and Quality Control**

Recruitment, health education, and telephone assessments will be conducted by trained bilingual (English/Spanish) health educators and assessors. We have built a training course based upon staff feedback and quality control during previous studies (The B.A.B.Y Study and Estudio VIDA) with an accompanying manual which includes instruction on motivational interviewing techniques, role-playing, specific study protocols and scripts. Regular quality assurance and quality control reviews will include observations of the health educator. The observations will be done by asking participants permission to audio record a contact. The recordings will be scheduled so that the same day as the contact, the study coordinator will be available to review the interaction with the health educator, assess the quality of the interaction, and use the time as an opportunity to re-train the health educator as necessary. Assessors will be monitored in the same way. The audio recordings will be (audibly) identified with the participant’s study ID and contain no PHI. The recordings will be destroyed once they are reviewed. When not in use, the recorder/memory card will be kept in a locked container in a locked room at Baystate. If a staff person is observed departing from standard protocol, feedback and recommendations for retraining will be provided. The assessor will be blinded to the treatment assignment. The health educator will be blinded to the results of the assessor’s assessments. The medical record abstractor will be trained using the Medical Record Abstraction Manual and training program developed as part of our prior studies. The medical record abstractor will be blinded to the treatment assignment.

Our research group has had extensive experience with Baystate medical records over the past 12 years. We have systematically assessed the records for organization as well as completeness through correlations with the hard copy medical record systems and the billing database. Key variables for abstraction have been found to be accurately reported on the electronic system. Reliability of medical record abstraction will be assessed by abstraction of 10% of the medical records twice. The abstractor will be retrained based upon the findings from this assessment. Monthly staff meetings will be attended by the telephone assessor, health educator, medical record abstractor, data analyst, laboratory technician, Project Manager and the Principal Investigator.

**D.15. Data Management**

All participants will be assigned a Study Identification code (sequentially numbered) which will be used for identification purposes.

Study personnel will have access, on an as-needed basis, to a password protected and encrypted linking file (stored on the network at Baystate and a secure server at the School of Public Health and Health Sciences (SPHHS) at UMASS Amherst) which will allow for the identification of participant by name and include contact information, data collection dates, date of last menstrual period (LMP), and blood specimen locators. This is necessary to allow for women to be identified and approached for follow-up visits and meetings, as well as to be assessed over the phone, and for chart review.

No participant data will be individually identified or released to anyone other than the study investigators.

Data collection, including medical record abstraction, will take place using a dedicated REDCap project on the UMASSMED REDCap server. This project will be controlled and maintained by the Project Manager. Only individuals on the approved Core Data form will have access to the REDCap project. Only the UMass Project Manager and UMass PI will have access to export data from the REDCap project. As required, the UMass Project Manager or PI will export data labeled with a study ID to allow for processing and storage in the secure UMass study database (see below). To enable data management the exported data will include dates, LMP or delivery date, for instance, however there will be no additional identifying information such as name, MRN, or address exported to the UMass database. Note: the REDCap project will not contain data from biomarker testing or activity monitor readout; these data will be merged with data exported from REDCap by UMass staff.

Research staff at University of California, San Diego (UCSD) will analyze coded responses from participants’ tailoring questionnaires using states of change analysis from the transtheoretical model. They will then provide UMass Amherst with the tailored intervention that is most appropriate for the participant. The communication between UMass Amherst and UCSD will be via email. UCSD research staff will not have access to any identifiable data.

Research staff at Northeastern University Dietary Assessment Center will enter data from the coded 24-hr diet recalls. They will then return the quantitative data to UMass Amherst. Communication between UMass Amherst and the study team at Northeastern will be via United States Postal Service and email. Northeastern research staff will not have access to any identifiable data.

Dr. Nader Rifal of Children’s Hospital Boston will receive specimens labeled by study ID only. Dr. Rifal will send results to Dr. Chasen-Taber’s team at UMass Amherst via email. Dr. Chasen-Taber’s team will store the lab data on secure School of Public Health and Health Sciences server with access restricted to the study team and will later merge the lab data with the data export from REDCap for analysis.

Data from Activity Monitors is downloaded at UMass Amherst. Activity Monitors are mailed by the study participant to UMass Amherst with an envelope insert with the study ID number, no name or other identifiers. The return address on the envelope is the OB/GYN Research Office at Baystate. Dr. Chasen-Taber’s team will store the activity monitor data on the secure School of Public Health and Health Sciences server with access restricted to the study team and will later merge the lab data with the data export from REDCap for analysis.

All other study information source documents that include identifying information (i.e. Informed Consent document, medical clearance form) will be secured in a locked filing cabinet in the locked Baystate Ob/Gyn office.

All passwords will be at least 8 characters long with at least one number and one capital letter.

Safeguards for Ineligible Subjects Cohort: Using the Potential Participants Form (PPF) a research assistant compiles a spreadsheet with the participants’ name, medical record number, date of screening and reason(s) for ineligibility. This file is password protected per Baystate policy and stored on the Baystate network in a shared folder with access restricted to IRB approved personnel only. After the study is completed, the reasons for ineligibility are counted and the de-identified results will be sent to UMASS and this list will be destroyed. Identified data from this list will never be sent outside of Baystate.

The databases will be maintained and managed by the Project Manager at UMass Amherst.

**D.16. Statistical Analysis**

Data from this study will be used to evaluate the impact of a Lifestyle Intervention on postpartum weight loss (Aim #1); postpartum biomarkers of insulin resistance (Aim #2) and other postpartum biomarkers of cardiovascular risk (Aim #3) among Hispanic women with a history of abnormal glucose tolerance in pregnancy.

The primary analysis will evaluate differences in the change from 6 wks postpartum to 12 mos postpartum between the groups (an intent to treat analysis) in postpartum weight loss (aim #1), biomarkers of insulin resistance (aim #2), and other postpartum biomarkers of CVD risk (aim #3). Initial analyses will describe the correlation of factors and investigate simple transformations for normality. The primary analysis will be based on a mixed model with random subject effects, including a common mean at baseline for the treatment groups, a period effect, and an intervention by period interaction.^149^ The intervention by period interaction is a measure of the intervention effect, and corresponds to the estimate of an intervention effect in an analysis of covariance. An advantage of this approach is that data can be included for women with only baseline measures (e.g., women who get pregnant during the follow-up period or who are lost to follow-up), which will help to reduce standard error estimates. Women who get pregnant during the follow up are removed from the study at the time of their positive pregnancy test. Their data will only be used in analysis of baseline data. The mixed model analysis will enable inclusion of time varying covariates such as breastfeeding behaviors, depression, sleep, which may vary between baseline and follow-up for subjects. Equivalence of the treatment groups will be assessed by comparing the distributions of the potential confounders between each group. In addition, we will investigate established risk factors for type 2 diabetes as potential confounders or effect modifiers. Generally, we will consider a change in the parameter estimate for the exposure (at least 15%), relative to that observed when not controlling for the variable as reflective of confounding. Effect modification will be evaluated by inspection of stratum specific odds ratios as well as by evaluating the statistical significance of interaction terms via chi-square likelihood ratio tests. Important potential effect modifiers include study site, pre-gravid BMI, and breastfeeding behaviors. In addition to evaluating the impact of study arm assignment on the outcomes of interest, we will also model the relationship between measured levels of exercise and diet with weight loss and with each biomarker, while adjusting for possible confounders using mixed effect linear regression models. Exercise and dietary measures will be modeled both continuously to determine whether there is a linear dose-response relationship with the outcomes as well as categorically to determine if there is threshold amount of activity or dietary factor which reduces risk. For the categorical analysis, we will determine whether to use quartiles or other cut-points based upon our examination of the distribution of scores. We plan to test hypotheses with two sided tests.

The study statistician will perform subanalyses in which they stratify participants according to their eligibility criteria (e.g., failing either a 1 hour and/or 3 hour GTT) to evaluate whether there is a different risk for eventually developing diabetes.

Data from this study will also be used to evaluate the impact of a Lifestyle Intervention on the adoption and maintenance of increased physical activity and reduction in total calories (Aim #4). Mixed model analyses will be used to account for the repeated measures of exercise and diet and will be used to evaluate adoption and maintenance of change in the Lifestyle intervention group. The mixed model will incorporate fixed assessment period effects, using the baseline measure as the reference and random subject effects, and will be fit and incorporate covariates as described for Aims #1-3. As a 2nd analysis we will compare adoption and maintenance of change in the intervention group vs. the control group by adding a fixed treatment group effect. Analyses will be performed for each assessment method (i.e., questionnaire, 24hr recall, accelerometer) and results will be compared.

Accounting for Missing Data: Although every effort is made to avoid missing data, participants with missing data on key variables will be compared to participants with complete data on patient baseline characteristics and outcome measures to describe potential bias due to differential loss of data. We also plan to use propensity score techniques^150-152^ to evaluate the potential sensitivity of results to missing at random assumptions. We will address the issue of multiple comparisons by carefully considering the biologic rationale of any observed associations.

**D.17. Power**

Power was calculated using NCSS PASS 2008 software. Standard deviations were based on prior studies conducted among Hispanic populations and postpartum populations by our research group and those of others.^1, 93, 102, 141, 153-156^ Calculations are for 2-sided tests with a significance level of alpha=5%. For multiple regression models, we assumed that covariates accounted for 20% of the variation. Table 4 below shows the smallest mean difference in change from baseline in the Lifestyle Intervention vs. the Health and Wellness Control arm that can be detected given a final sample size of n=128 in each arm at 80% power. Cohen^157^ defines a detectable difference of 0.2 standard deviations as a “small” effect size and 0.5 standard deviations as a “medium” effect size. The detectable mean differences in Table 4 are equivalent to a range of 0.35 to 0.40 standard deviations, or a “small-medium” effect size. For example, we have 80% power to detect as small as a 2.6% difference in percent change in HbA1c between treatment arms. By way of comparison, our prior trial among Hispanics observed a 7.1% difference in percent change in HbA1c between the intervention and control arms (-9.9% in intervention vs. -2.8% in controls, p=0.03)93 demonstrating that these differences are within the capability of the lifestyle intervention. We will have greater power to detect greater differences.

**D.18. Environment**

In brief, Baystate Medical Center, the recruitment site, is the major medical resource in Western Massachusetts and the third largest acute-care hospital in New England, with 725 beds currently in service and approximately 4,500 deliveries per year. The School of Public Health and Health Sciences at UMASS maintains a micro computer research room for faculty, staff and students. Fifteen PC compatible microcomputers, a file server, a scanner and two laser printers are connected to the Department's Local Area Network (LAN). The LAN enables secure hard disk space for research projects that is accessible by multiple users and backed-up daily. Dr. Nader Rifai’s (Department of Laboratory Medicine, Harvard University) lab at Children’s Hospital, Boston, MA is a fully equipped laboratory with all of the requisite centrifuges, water baths, pipetters, etc. required to do the hormonal assays.

**D.19. Timeline**

The timeline takes into account adequate time for study start up (3 mos), 3.3 years (40 months) of recruitment, and follow-up of the last remaining participant (14 mos). Data analysis will be ongoing and the last 3 months of the study will be dedicated to final analyses and manuscript writing for a total of 60 mos (5 year study).


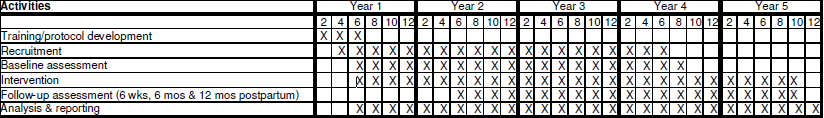


**D.20. Limitations and Alternatives**

We chose not to utilize a group-based intervention. Prior reviews indicate that the time and child care pressures that postpartum women report as barriers to physical activity indicate that the requirement to attend groups at scheduled times and to travel to and from venues would deter many.^130, 131^ Indeed, retention levels in supervised group physical activity programs have been observed to fall to ~50% after 6 months.^131, 132^ In contrast, our research group,^85^ as well as others,^85, 133, 134^ have found that individually-tailored lifestyle interventions delivered in person, via telephone, and via mail produce greater or comparable changes in behavior at a more cost efficient level compared with group-based interventions.

**E. Protection of Human Subjects**

**1. Risks to the Subjects**

**Human Subjects Involvement and Characteristics:**

The population under study includes 300 pregnant/postpartum women between the ages of 16 and 45 years being seen for prenatal care at Baystate Medical Center or UMass Medical Center. All participants will be Hispanic. Women are healthy and are considered ineligible if they have 1) history of type 1 or type 2 diabetes, heart disease, or chronic renal disease, 2) contraindications to postpartum participation in moderate physical activity or a low-fat/high-fiber diet (e.g., Crohn’s disease, ulcerative colitis), 3) inability to read English or Spanish at a 6th grade level, or 4) <16 or >45 yrs of age. Women who have a preterm birth after enrollment or a stillbirth will not be excluded as the focus is on maternal postpartum risk for subsequent chronic disease. Women carrying multiples will be excluded, as will women who are greater than 30 weeks gestational age.

#### Sources of Materials:

At the time of recruitment (24-28 weeks gestation), a blood sample to evaluate serum biomarkers associated with insulin resistance and other cardiovascular risk factors will be collected (termed “blood draw”). A total 700 microliters (uL) of serum will be collected which is equivalent to 1.5 milliliters (mL) or less than a teaspoon of whole blood. Physical activity, diet, and other covariates will also be assessed at recruitment. Subsequent assessments will be conducted at 6-weeks postpartum, 6-months, and 12-months postpartum. A urine pregnancy test will also be performed at 6-weeks postpartum, 6-months and 12-months postpartum. Women who are identified as having a positive pregnancy test will be removed from the study at the time of their positive pregnancy test and referred to their obstetric care provider. Measurements of compliance with exercise and diet will include activity monitors (worn for a 4-day period within each of these assessment time points), tracking the number of intervention sessions attended, number of booster calls participated in, number of assessments (e.g., questionnaires, blood samples) completed, as well as an assessment of degree of participation in physical activity and healthy dietary behaviors. No participants will be excluded from the study based on noncompliance. Assessments to measure other important covariates will also be collected at these assessment time points. Subjects will be under no obligation to complete the assessments, activity monitoring, or blood collections. Pregnancy and postpartum medical and laboratory records will be reviewed for obstetric and medical history, clinical characteristics of the index pregnancy, and laboratory results. All data will be used specifically for research purposes.

#### Potential Risks:

The exercise intervention focuses on increasing walking and developing a more active lifestyle as per recommendations of the American College of Obstetricians and Gynecologists for postpartum women (ACOG Committee opinion. January 2002: “Exercise during Pregnancy and the Postpartum Period”) and the American Diabetes Association guidelines for women with previous GDM (The American College of Sports Medicine and the American Diabetes Association Joint Position Statement: "Exercise and Type 2 Diabetes"). These recommendations include 30 minutes or more of moderate exercise a day on most, if not all, days of the week. The overall goal of the dietary intervention is to reduce total calories via a diet with lower intake of calorie dense foods (e.g., fast food, high-fat snacks, fried foods and sugar-sweet beverages), saturated fat, and higher fruit and vegetable intake as recommended by the American Diabetes Association.

A possible risk in this study involves the social/psychological risk for an individual resulting from inadvertent disclosure of confidential medical history information. Confidentiality of the data will be ensured by assigning a subject identification number to each participant. All data collected in the study will be entered by subject identification number only into an encrypted, password protected, firewall protected server located at UMASS Amherst. No participant data will be individually identified or released to anyone other than the study investigators. Questionnaires, identified by study number only, will be kept in a locked filing system separate from the name-address file of participants in the study. Only the Principal Investigator and the Project Manager will have access to the data in its raw state. All other authorized study staff will view coded data via forms and reports created by the data manager

Collection of blood specimen: Serum will be collected at each assessment time point. For most people, needle punctures to obtain blood specimens do not cause any serious problems. However, needle punctures may cause bleeding, bruising, discomfort, infections, dizziness, or fainting. To reduce this concern, the sample will be collected at a Baystate Reference Laboratory. A total 700 microliters (uL) of serum will be collected which is equivalent to 1.5 milliliters (mL) or less than a teaspoon of whole blood.

**2. Adequacy of Protection Against Risks**

**Recruitment and Informed Consent:**

A bilingual and bicultural recruiter will describe the study and its relevance to patients at the time of routine

screening for GDM. Patients will be told that they are under no obligation to participate in this study. Those

who agree will complete a written informed consent which describes the: purpose of research, procedures,

risks and discomforts, benefits, costs & compensation, alternatives to participation, patient enrollment/length of

study, confidentiality, voluntary participation, requests for additional information, and voluntary consent.

**Protection Against Risk:**

Prior to randomization (~29 wks gestation), during pregnancy, and again after delivery, participants will fill out the Physical Activity Readiness Questionnaire. If they answer yes to any of the questions on the questionnaire, Dr. Markenson (Co-Investigator, and Director of Maternal/Fetal Medicine at Baystate) or Dr. Moore Simas (Co-Investigator, UMASS Medical Center Worcester) will evaluate the participant for study inclusion by reviewing prenatal visit records, the delivery medical records, and/or postpartum visit records. It is important to note that we conservatively scheduled the onset of the Active Intervention Phase to not begin until 6 weeks postpartum, a time by which the physiologic and morphologic changes of pregnancy no longer persist for the majority of women (ACOG Committee opinion. January 2002: “Exercise during Pregnancy and the Postpartum Period”). The American College of Obstetricians and Gynecologists recommends that, in the absence of complications from delivery, a mild exercise program consisting of walking, pelvic floor exercises, and light stretching of all muscle groups can begin in the immediate postpartum period (ACOG Committee opinion. January 2002: “Exercise during Pregnancy and the Postpartum Period”). In the case that Drs. Markenson and Moore Simas determine there are contraindications to the lifestyle intervention, the woman will be excluded from the study and immediately contacted by the Project Coordinator by telephone and in writing.

Although the likelihood is low, our research team has well-established procedures for monitoring and responding to adverse events resulting from moderate intensity physical activity. First, during the orientation participants will be provided with contact information for study personnel and instructed to contact our team if any exercise related adverse event occurs (e.g., soft tissue injury). Additionally, we will be corresponding with the study participants over brief time periods to obtain study-related questionnaires. As part of those questionnaires, participants will be asked whether any adverse event occurred. In the case of any report, the Principal Investigator will be immediately notified, who will then notify the DSMB and Institutional Review Board. In cases of injuries that interfered with functioning (i.e., ability to move about), the participant will be asked to cease activity and obtain physician clearance before continuing.

Studies have shown the safety and efficacy of moderate exercise and weight reduction during lactation without affecting infant growth, milk composition (i.e., immunological component concentrations, vitamin B6, essential fatty acids), or milk volume (ACOG Committee Opinion No. 361: “Breastfeeding: Maternal and Infant Aspects”). Moderate weight reduction while nursing is safe and does not compromise neonatal weight gain (ACOG Committee opinion. January 2002: “Exercise during Pregnancy and the Postpartum Period”). Health educators will encourage lactating women to follow recommended guidelines of the American College of Obstetricians and Gynecologists which include exercising after the baby has been fed or the breasts are empty to reduce discomfort; use of a good support bra and avoidance of a sports bra because of breast compression; and maintenance of adequate nutrition and hydration to support the energy demands of breastfeeding and exercise (ACOG Committee opinion. January 2002: “Exercise during Pregnancy and the Postpartum Period”).

**3. Potential Benefits of the Proposed Research to the Subjects and Others**

The potential benefit to society of this study could be substantial in recognizing exercise, diet, as a tool to prevent risk factors for type 2 diabetes and cardiovascular disease among postpartum women with a history of GDM diagnosis. An additional benefit to study participants is postpartum glucose screening which is often not performed in women with a history of GDM. By participating in this study, participants will receive information about how to become more physically active, *reduce caloric intake*, and reduce postpartum weight retention that may lead to improved wellness. Those in the Health & Wellness comparison group will receive self-help material currently available to the public on topics including stress management, cancer prevention, and alternative medicine.

**4. Importance of the Knowledge to Be Gained**

The importance of the study lies in the fact that changes in modifiable risk factors may reduce the risk of subsequent type 2 diabetes and cardiovascular disease. The intervention protocol can readily be translated into clinical practice in underserved and minority populations. Indeed, the impact of such postpartum lifestyle modifications is likely to be greatest in ethnicities, such as Hispanics, with consistently high rates of GDM and low rates of physical activity. The risks to participants in this study are judged to be minor. The anticipated benefits are great, insofar as the results will be used to further understand the unique factors related to promoting physical activity adoption and maintenance among Latinas among whom health disparities and associated sedentary lifestyles remain an enormous public health concern.

**5. Data Safety and Monitoring Plan**

This trial will be monitored in compliance with an independent Data and Safety Monitoring Board (DSMB) written in concordance with the guidelines from the National Institutes of Health. The DSMB will: 1) review the research protocol and plans for data safety and monitoring, 2) evaluate the progress of the trial with biannual assessments of data quality and timeliness, participant recruitment, accrual and retention, participant risk versus benefit, and reports from related studies, and 3) make recommendations to the IRB and investigators concerning continuation or conclusion of the trial. The DSMB will consist of three members including a physician, Dr. Ron Burkman, who will serve as Chair, a statistician (to be named), and Dr Priscilla Clarkson as an outside researcher at UMASS Amherst all of whom will be knowledgeable about the study’s content but not directly involved in the study nor in a supervisory role to study personnel. The DSMB will meet biannually. Prior to each meeting, the Principal Investigator will submit a report which will include: 1) safety of the protocol participants, specifically if any adverse events have occurred;

2) validity and integrity of the data; 3) enrollment rate relative to expectation; 4) retention of participants; 5) data completeness; and 6) preliminary data analysis. The principal investigator will receive a written response regarding the DSMB’s approval or suspension of the study. The report will be forwarded to the Institutional Review board. Monitoring activities by the Principal Investigator and the DSMB will continue until all participants have completed the study and are beyond the time point at which study-related adverse events would presumably be encountered.

# Women and Minority Inclusion in Clinical Research

The population under study includes 300 postpartum women between the ages 16 and 45 years being seen for prenatal care at Baystate Medical Center and UMass Worcester Medical Center. The study is a postpartum intervention among those diagnosed with abnormal glucose tolerance of pregnancy; therefore all subjects are women.

All women are Hispanic. Hispanic women are the fastest growing minority group in the U.S. and have the highest rates of sedentary behavior as well as postpartum diabetes after a diagnosis of gestational diabetes. Hispanics have, overall, been underrepresented in prior research.


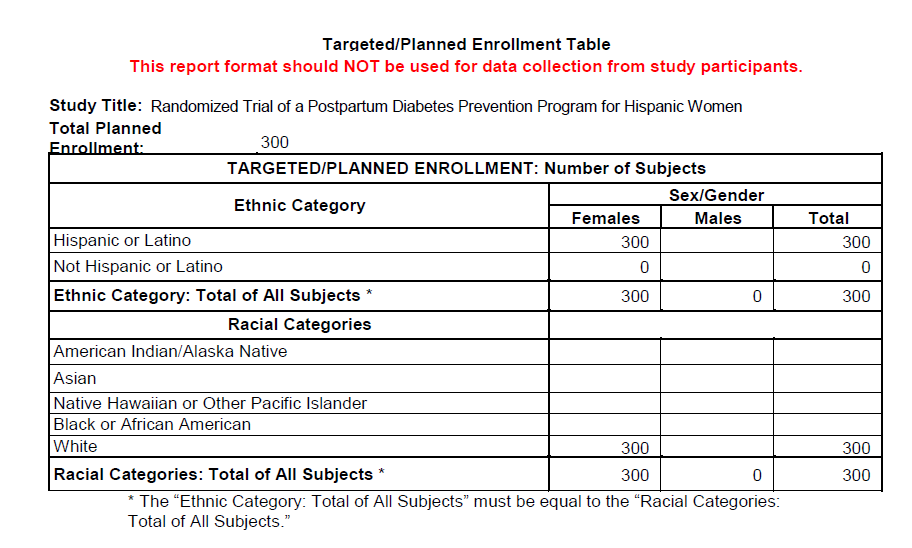


**Inclusion of Children:**

Children between the ages of 16 and 21 will be included. Children younger than 16 will not be included for 2 reasons: 1) the study is a postpartum intervention among women with a history of abnormal glucose tolerance pregnancy, 2) modifiable determinants of type 2 diabetes and cardiovascular disease may differ substantively among children under age 16 therefore precluding direct applicability of hypotheses to this age group. Note: Studies support the lowering of the eligibility age, such as [Paediatr Drugs.](http://www.ncbi.nlm.nih.gov/pubmed/11960510) 2002;4(4):209-21.

Increasing incidence of type 2 diabetes in children and adolescents: treatment considerations.

**F. Vertebrate Animals**

None

**G. Literature Cited**

1. O'Toole ML, Sawicki MA, Artal R. Structured diet and physical activity prevent postpartum weight

retention. J Womens Health (Larchmt) 2003;12(10):991-8.

2. Lakshman R, Landsbaugh J, Schiff A, Hardeman W, Ong K, Griffin S. Development of a questionnaire to

assess maternal attitudes towards infant growth and milk feeding practices. The international journal of

behavioral nutrition and physical activity 2011;8(1):35.

3. Wild S, Roglic G, Green A, Sicree R, King H. Global prevalence of diabetes: estimates for the year 2000

and projections for 2030. Diabetes Care 2004;27(5):1047-53.

4. Kim C, Newton KM, Knopp RH. Gestational diabetes and the incidence of type 2 diabetes: a systematic

review. Diabetes Care 2002;25(10):1862-8.

5. Retnakaran R. Glucose tolerance status in pregnancy: a window to the future risk of diabetes and

cardiovascular disease in young women. Current diabetes reviews 2009;5(4):239-44.

6. Bellamy L, Casas J, Hingorani A, Williams D. Type 2 diabetes mellitus after gestational diabetes: a

systematic review and meta-analysis. The Lancet 2009;373(9677):1773-9.

7. Cheung NW, Byth K. Population health significance of gestational diabetes. Diabetes Care

2003;26(7):2005-9.

8. Kjos SL, Peters RK, Xiang A, Henry OA, Montoro M, Buchanan TA. Predicting future diabetes in Latino

women with gestational diabetes. Utility of early postpartum glucose tolerance testing. Diabetes

1995;44(5):586-91.

9. Buchanan TA, Xiang A, Kjos SL, Lee WP, Trigo E, Nader I et al. Gestational diabetes: antepartum

characteristics that predict postpartum glucose intolerance and type 2 diabetes in Latino women. Diabetes

1998;47(8):1302-10.

10. Retnakaran R, Qi Y, Connelly P, Sermer M, Hanley A, Zinman B. Risk of Early Progression to Prediabetes

or Diabetes in Women with Recent Gestational Dysglycemia but Normal Glucose Tolerance at 3-

months Postpartum. Clin Endocrinol (Oxf) 2010;:Jun 9. [Epub ahead of print].

11. Kieffer E, Sinco B, Kim C. Health behaviors among women of reproductive age with and without a

history of gestational diabetes mellitus. Diabetes Care 2006;29(8):1788-93.

12. Ratner RE, Christophi CA, Metzger BE, Dabelea D, Bennett PH, Pi-Sunyer X et al. Prevention of

diabetes in women with a history of gestational diabetes: effects of metformin and lifestyle interventions. J Clin

Endocrinol Metab 2008;93(12):4774-9.

13. Hamman R, Wing R, Edelstein S, Lachin J, Bray G, Delahanty L et al. Effect of weight loss with lifestyle

intervention on risk of diabetes. Diabetes Care 2006;29(9):2102-7.

14. Kim C. Gestational diabetes: risks, management, and treatment options. Int J Womens Health

2010;2:339-51.

15. Lovelady CA, Garner KE, Moreno KL, Williams JP. The effect of weight loss in overweight, lactating

women on the growth of their infants. N Engl J Med 2000;342(7):449-53.

16. Lovelady CA, Nommsen Rivers LA, McCrory MA, Dewey KG. Effects of exercise on plasma lipids and

metabolism of lactating women. Med Sci Sports Exerc 1995;27(1):22

17. Norris SL, Zhang X, Avenell A, Gregg E, Schmid CH, Lau J. Long-term non-pharmacological weight loss

interventions for adults with prediabetes. Cochrane database of systematic reviews 2005;(2):CD005270.

18. Moore H, Summerbell C, Hooper L, Cruickshank K, Vyas A, Johnstone P et al. Dietary advice for

treatment of type 2 diabetes mellitus in adults. Cochrane database of systematic reviews 2004;(3):CD004097.

19. Shaw K, O'Rourke P, Del Mar C, Kenardy J. Psychological interventions for overweight or obesity.

Cochrane database of systematic reviews 2005;(2):CD003818.

20. Buchanan TA, Xiang AH, Peters RK, Kjos SL, Marroquin A, Goico J et al. Preservation of pancreatic

beta-cell function and prevention of type 2 diabetes by pharmacological treatment of insulin resistance in highrisk hispanic women. Diabetes 2002;51(9):2796-803.

21. Stuebe A, Ecker J, Bates DW, Zera C, Bentley-Lewis R, Seely E. Barriers to Follow-up for Women with a

History of Gestational Diabetes. Am J Perinatol 2010;:705-10.

22. Miller G, Nicklas B, Loeser R. Inflammatory biomarkers and physical function in older, obese adults with

knee pain and self-reported osteoarthritis after intensive weight-loss therapy. J Am Geriatr Soc

2008;56(4):644-51.

23. Kim S, Jung I, Kim J. Exercise reduces C-reactive protein and improves physical function in automotive

workers with low back pain. J Occup Rehabil 2008;18(2):218-22.

24. Puglisi M, Fernandez M. Modulation of C-reactive protein, tumor necrosis factor-alpha, and adiponectin

by diet, exercise, and weight loss. J Nutr 2008;138(12):2293-6.

25. Puglisi M, Vaishnav U, Shrestha S, Torres-Gonzalez M, Wood R, Volek J et al. Raisins and additional

walking have distinct effects on plasma lipids and inflammatory cytokines. Lipids in Health and Disease

2008;7:14.

26. Kriketos AD, Gan SK, Poynten AM, Furler SM, Chisholm DJ, Campbell LV. Exercise increases

adiponectin levels and insulin sensitivity in humans. Diabetes Care 2004;27(2):629-30.

27. Hulver MW, Zheng D, Tanner CJ, Houmard JA, Kraus WE, Slentz CA et al. Adiponectin is not altered

with exercise training despite enhanced insulin action. Am J Physiol Endocrinol Metab 2002;283(4):E861-5.

28. Kraemer RR, Aboudehen KS, Carruth AK, Durand RT, Acevedo EO, Hebert EP et al. Adiponectin

responses to continuous and progressively intense intermittent exercise. Med Sci Sports Exerc

2003;35(8):1320-5.

29. Castellano V, Patel D, White L. Cytokine responses to acute and chronic exercise in multiple sclerosis. J

Appl Physiol 2008;104(6):1697-702.

30. Campbell K, Campbell P, Ulrich C, Wener M, Alfano C, Foster-Schubert K et al. No reduction in Creactive

protein following a 12-month randomized controlled trial of exercise in men and women. Cancer

epidemiology, biomarkers prevention 2008;17(7):1714-8.

31. Zern T, Wood R, Greene C, West K, Liu Y, Aggarwal D et al. Grape polyphenols exert a cardioprotective

effect in pre- and postmenopausal women by lowering plasma lipids and reducing oxidative stress. J Nutr

2005;135(8):1911-7.

32. Sharman M, Volek J. Weight loss leads to reductions in inflammatory biomarkers after a very-lowcarbohydrate diet and a low-fat diet in overweight men. Clin Sci 2004;107(4):365-9.

33. Reseland JE, Anderssen SA, Solvoll K, Hjermann I, Urdal P, Holme I et al. Effect of long-term changes

in diet and exercise on plasma leptin concentrations. Am J Clin Nutr 2001;73(2):240-5.

34. Koutsari C, Karpe F, Humphreys SM, Frayn KN, Hardman AE. Plasma leptin is influenced by diet

composition and exercise. Int J Obes Relat Metab Disord 2003;27(8):901-6.

35. Stefan N, Fritsche A, Weikert C, Boeing H, Joost H, Hring H et al. Plasma fetuin-A levels and the risk of

type 2 diabetes. Diabetes 2008;57(10):2762-7.

36. Stefan N, Hennige A, Staiger H, Machann J, Schick F, Krber S et al. Alpha2-Heremans-Schmid

glycoprotein/fetuin-A is associated with insulin resistance and fat accumulation in the liver in humans. Diabetes

Care 2006;29(4):853-7.

37. Orozco L, Buchleitner A, Gimenez-Perez G, Roqué I Figuls M, Richter B, Mauricio D. Exercise or

exercise and diet for preventing type 2 diabetes mellitus. Cochrane database of systematic reviews

2008;(3):CD003054.

38. Nield L, Summerbell C, Hooper L, Whittaker V, Moore H. Dietary advice for the prevention of type 2

diabetes mellitus in adults. Cochrane database of systematic reviews 2008;(3):CD005102.

39. Thomas DE, Elliott EJ, Naughton GA. Exercise for type 2 diabetes mellitus. Cochrane database of

systematic reviews 2006;3:CD002968.

40. U.S. Department of Commerce Economics and Statistics Administration. US Census Bureau. The

American Community—Hispanics: 2004. 2007.

41. Misra D. The Women's Health Data Book (3rd ed.). Washington, DC: Jacob's Women's Health Institute;

2001.

42. Giardina E, Laudano M, Hurstak E, Saroff A, Fleck E, Sciacca R et al. Physical activity participation

among Caribbean Hispanic women living in New York: relation to education, income, and age. Journal of

women's health 2009;18(2):187-93.

43. Zambrana RE, Logie LA. Latino child health: need for inclusion in the US national discourse. Am J Public

Health 2000;90(12):1827-33.

44. Centers for Disease Control and Prevention (CDC). Prevalence of physical activity, including lifestyle

activities among adults--United States, 2000-2001. MMWR Morb Mortal Wkly Rep 2003;52(32):764-9.

45. Anonymous . Health disparities experienced by Hispanics--United States. Morb Mortal Weekly Rep

2004;53(40):935-7.

46. Stasenko M, Cheng YW, McLean T, Jelin AC, Rand L, Caughey AB. Postpartum Follow-up for Women

with Gestational Diabetes Mellitus. Am J Perinatol 2010;:737-42.

47. Flegal KM, Ezzati TM, Harris MI, Haynes SG, Juarez RZ, Knowler WC et al. Prevalence of diabetes in

Mexican Americans, Cubans, and Puerto Ricans from the Hispanic Health and Nutrition Examination Survey,

1982-1984. Diabetes Care 1991;14(7):628-38.

48. Centers for Disease Control and Prevention. Age-adjusted percentage of civilian, noninstitutionalized

population with diagnosed diabetes, Hispanics, United States, 1980-2007. 7/29/09.

49. Hajat A, Lucas JB, Kington R. Health outcomes among Hispanic subgroups: data from the National

Health Interview Survey, 1992-95. Adv Data 2000;(310)(310):1-14.

50. Himmelgreen DA, Perez-Escamilla R, Peng Y, Angela B. Birthplace, length of time in the U.S., and

language are associated with diet among inner-city Puerto Rican women. Ecology of Food and Nutrition

2005;44:105-122.

51. Kim C. Managing women with gestational diabetes mellitus in the postnatal period. Diabetes, obesity

and metabolism 2010;12(1):20-5.

52. Frayne SM, Burns RB, Hardt EJ, Rosen AK, Moskowitz MA. The exclusion of non-English-speaking

persons from research. Journal of general internal medicine 1996;11(1):39-43.

53. Durant R, Davis R, St George DMM, Williams I, Blumenthal C, Corbie-Smith G. Participation in research

studies: factors associated with failing to meet minority recruitment goals. Ann Epidemiol 2007;17(8):634-42.

54. Chasan-Taber L, Fortner RT, Hastings V, Markenson G. Strategies for recruiting Hispanic women into a

prospective cohort study of modifiable risk factors for gestational diabetes mellitus. BMC Pregnancy Childbirth

2009;9:57.

55. Marquez D, Bustamante E, Bock B, Markenson G, Tovar A, Chasan-Taber L. Perspectives of Latina and

non-Latina white women on barriers and facilitators to exercise in pregnancy. Women Health 2009;49(6):505-

21.

56. Tovar A, Must A, Bermudez OI, Hyatt R, Chasan-Taber L. The Impact of Gestational Weight Gain and

Diet on Abnormal Glucose Tolerance During Pregnancy in Hispanic Women. Maternal and child health journal

2009;13(4):520-530.

57. Tovar A, Chasan-Taber L, Bermudez O, Hyatt R, Must A. Knowledge, Attitudes, and Beliefs Regarding

Weight Gain During Pregnancy Among Hispanic Women. Matern Child Health J 2009;:[Epub ahead of print].

58. Chasan-Taber L, Fortner R, Gollenberg A, Buonnaccorsi J, Dole N, Markenson G. A prospective cohort

study of modifiable risk factors for gestational diabetes among Hispanic women: design and baseline

characteristics. Journal of women's health 2010;19(1):117-124.

59. Chasan-Taber L. Emerging Science: Interventions in Women at Risk for GDM during Pregnancy. In: C.

Kim, A. Ferrara eds. Gestational Diabetes During and After Pregnancy. London: Springer-Verlag; 2010.

60. Fortner R, Pekow P, Dole N, Markenson G, Chasan-Taber L. Risk Factors for Prenatal Depressive

Symptoms Among Hispanic Women. Matern Child Health J 2010; In Press.

61. Fortner R, Pekow P, Whitcomb B, Sievert L, Markenson G, Chasan-Taber L. Physical Activity and

Hypertensive Disorders of Pregnancy Among Hispanic Women. Med Sci Sports Exerc 2011 Apr;43(4):639-46.

62. Chasan-Taber L, Evenson KR, Sternfeld B, Kengeri S. Assessment of Recreational Physical Activity

During Pregnancy in Epidemiologic Studies of Birthweight and Length of Gestation: Methodologic Aspects.

Women & Health 2007;45(4):85-107.

63. Chasan-Taber L, Freedson PS, Roberts DE, Schmidt MD, Fragala MS. Energy expenditure of selected

household activities during pregnancy. Research Quarterly for Exercise and Sport 2007;78(1):133-137.

64. Chasan-Taber L, Schmidt MD, Pekow P, Sternfeld B, Manson JE, Solomon CG et al. Physical activity

and gestational diabetes mellitus among Hispanic women. Journal of women's health 2008;17(6):999-1008.

65. Chasan-Taber L, Schmidt M, Pekow P, Sternfeld B, Solomon C, Markenson G. Predictors of excessive

and inadequate gestational weight gain in Hispanic women. Obesity 2008;16(7):1657-66

66. Gollenberg A, Pekow P, Bertone-Johnson E, Freedson P, Markenson G, Chasan-Taber L. Physical

Activity and Risk of Small-for-Gestational-Age Birth Among Predominantly Puerto Rican Women. Matern Child

Health J 2011 Jan;15(1):49-59.

67. Gollenberg A, Pekow P, Bertone-Johnson E, Freedson P, Markenson G, Chasan-Taber L. Sedentary

behaviors and abnormal glucose tolerance among pregnant Latina women. Med Sci Sports Exerc

2010;42(6):1079-85.

68. Gollenberg A, Pekow P, Markenson G, Tucker K, Chasan-Taber L. Dietary behaviors, physical activity,

and cigarette smoking among pregnant Puerto Rican women. Am J Clin Nutr 2008;87(6):1844-51.

69. Gawade P, Pekow P, Markenson G, Plevyak M, Goh W, Chasan-Taber L. Physical activity before and

during pregnancy and duration of second stage of labor among Hispanic women. J Reprod Med

2009;54(7):429-35.

70. Fortner R, Pekow P, Solomon C, Markenson G, Chasan-Taber L. Prepregnancy body mass index,

gestational weight gain, and risk of hypertensive pregnancy among Latina women. Obstet Gynecol

2009;200(2):167.e1,167.e7.

71. Haskins A, Mukhopadhyay S, Pekow P, Markenson G, Bertone-Johnson E, Carbone E et al. Smoking

and risk of preterm birth among predominantly Puerto Rican women. Ann Epidemiol 2008;18(6):440-6.

72. Haskins A, Bertone-Johnson E, Pekow P, Carbone E, Fortner R, Chasan-Taber L. Smoking during

pregnancy and risk of abnormal glucose tolerance: a prospective cohort study. BMC Pregnancy and Childbirth

2010;10(1):55.

73. Detjen MG, Nieto FJ, Trentham-Dietz A, Fleming M, Chasan-Taber L. Acculturation and cigarette

smoking among pregnant Hispanic women residing in the United States. Am J Public Health

2007;97(11):2040-7.

74. Smirnakis K, Chasan-Taber L, Wolf M, Markenson G, Ecker J, Thadhani R. Postpartum diabetes

screening in women with a history of gestational diabetes. Obstet Gynecol 2005;106(6):1297-303.

75. Colberg-Ochs SR, Albright AL, Blissmer BJ, Braun B, Chasan-Taber L, Fernhall B et al. The American

College of Sports Medicine and the American Diabetes Association joint position statement. "Exercise and

Type 2 Diabetes." Medicine and science in sports and exercise 2010 Dec;42(12):2282-303.

76. Insaf T, Fortner R, Pekow P, Dole N, Markenson G, Chasan-Taber L. Prenatal Stress, Anxiety, and

Depressive Symptoms as Predictors of Intention to Breastfeed among Hispanic Women. J Women's Health [In

Press].

77. Schmidt MD, Freedson PS, Pekow P, Roberts D, Sternfeld B, Chasan-Taber L. Validation of the Kaiser

Physical Activity Survey in pregnant women. Med Sci Sports Exerc 2005;38(1):42-50.

78. Chasan-Taber L, Schmidt MD, Pekow P, Sternfeld B, Manson J, Markenson G. Correlates of Physical

Activity in Pregnancy among Latina Women. Matern Child Health J 2007;11(4):353-63.

79. Chasan-Taber L. An Expert's View: Gestational Diabetes and Exercise. In: Deschenes M, Kraemer B,

Fleck S. Exercise Physiology. Philadelphia, PA: Lippincott Williams & Wilkins; In Press.

80. Tovar A, Chasan-Taber L, Eggleston E, Oken E. Postpartum Screening for Diabetes among Women

with a History of Gestational Diabetes Mellitus. Preventing Chronic Disease [In Press].

81. Dempsey JC, Butler CL, Williams MA. No need for a pregnant pause: physical activity may reduce the

occurrence of gestational diabetes mellitus and preeclampsia. Exerc Sport Sci Rev 2005;33(3):141-9.

82. Tieu J. Dietary advice in pregnancy for preventing gestational diabetes mellitus. The Cochrane database

of systematic reviews 2008;(2):CD006674.

83. Leiferman JA, Evenson KR. The effect of regular leisure physical activity on birth outcomes. Matern

Child Health J 2003;7(1):59-64.

84. Pereira MA, Rifas-Shiman SL, Kleinman KP, Rich-Edwards JW, Peterson KE, Gillman MW. Predictors of

change in physical activity during and after pregnancy: Project Viva. American journal of preventive medicine

2007;32(4):312-319.

85. Marcus BH, Lewis BA, Williams DM, Dunsiger S, Jakicic JM, Whiteley JA et al. A comparison of Internet

and print-based physical activity interventions. Arch Intern Med 2007;167(9):944-9.

86. Marcus BH, Napolitano MA, King AC, Lewis BA, Whiteley JA, Albrecht A et al. Telephone versus print

delivery of an individualized motivationally tailored physical activity intervention: Project STRIDE. Health

Psychol 2007;26(4):401-9.

87. Marcus BH, Bock BC, Pinto BM, Forsyth LH, Roberts MB, Traficante RM. Efficacy of an individualized,

motivationally-tailored physical activity intervention. Ann Behav Med 1998;20(3):174-80.

88. Marcus BH, Emmons KM, Simkin-Silverman LR, Linnan LA, Taylor ER, Bock BC et al. Evaluation of

motivationally tailored vs. standard self-help physical activity interventions at the workplace. Am J Health

Promot 1998;12(4):246-53.

89. Chasan-Taber L, Silveira M, Marcus BH, Stanek E, Ciccolo JT, Markenson G. Feasibility and Efficacy of

a Physical Activity Intervention among Pregnant Women: The Behaviors Affecting Baby and You (B.A.B.Y.)

Study. Journal of Physical Activity and Public Health [Under Review].

90. Pekmezi D, Neighbors C, Lee C, Gans K, Bock B, Morrow K et al. A culturally adapted physical activity

intervention for Latinas: a randomized controlled trial. Am J Prev Med 2009;37(6):495-500.

91. King AC, Haskell WL, Young DR, Oka RK, Stefanick ML. Long-term effects of varying intensities and

formats of physical activity on participation rates, fitness, and lipoproteins in men and women aged 50 to 65

years. Circulation 1995;91(10):2596-604.

92. Rosal MC, Olendzki B, Reed GW, Gumieniak O, Scavron J, Ockene I. Diabetes self-management

among low-income Spanish-speaking patients: a pilot study. Annals of behavioral medicine 2005;29(3):225-

235.

93. Merriam P, Tellez T, Rosal M, Olendzki B, Ma Y, Pagoto S et al. Methodology of a diabetes prevention

translational research project utilizing a community-academic partnership for implementation in an underserved

Latino community. BMC Medical research methodology 2009;9:20.

94. Ockene IS, Tellez TL, Rosal MC, Reed GW, Mordes J, Merriam PA et al. Outcomes of a Latino

community-based intervention for the prevention of diabetes: The Lawrence Latino Diabetes Prevention

Project. American Journal of Public Health [In Press].

95. Knowler WC, Barrett-Connor E, Fowler SE, Hamman RF, Lachin JM, Walker EA et al. Reduction in the

incidence of type 2 diabetes with lifestyle intervention or metformin. N Engl J Med 2002;346(6):393-403.

96. Rosal MC, Benjamin EM, Pekow PS, Lemon SC, von Goeler D. Opportunities and challenges for

diabetes prevention at two community health centers. Diabetes care 2008;31(2):247-54.

97. Rosal MC, Carbone ET, Goins KV. Use of cognitive interviewing to adapt measurement instruments for

low-literate Hispanics. The Diabetes educator 2003;29(6):1006-17.

98. Carbone ET, Rosal MC, Torres MI, Goins KV, Bermudez OI. Diabetes self-management: perspectives of

Latino patients and their health care providers. Patient Education and Counseling 2007;66(2):202-210.

99. von Goeler DS, Rosal MC, Ockene JK, Scavron J, De Torrijos F. Self-management of type 2 diabetes: a

survey of low-income urban Puerto Ricans. The Diabetes educator 2003;29(4):663-672.

100. Carbone ET, Lennon KM, Torres MI, Rosal MC. Testing the feasibility of an interactive learning styles

measure for U.S. Latino adults with type 2 diabetes and low literacy. International quarterly of community

health education 25(4):315-335.

101. Rosal M, Ockene I, Restrepo A, White M, Borg A, Olendzki B et al. Randomized Trial of a Literacy-

Sensitive, Culturally Tailored Diabetes Self-Management Intervention for Low-Income Latinos: Latinos en

Control. Diabetes Care 2011; Apr;34(4):838-44.

102. Rosal MC, Lemon SC, Nguyen OH, Driscoll NE, DiTaranto L. A methodological framework for adapting

weight loss interventions for real world implementation. Translational Behavioral Medicine [In Press].

103. Anonymous . Standards of medical care in diabetes--2009. Diabetes Care 2009;32 Suppl 1:S13-61.

104. HAPO Study Cooperative Research Group, Metzger BE, Lowe LP, Dyer AR, Trimble ER, Chaovarindr

U et al. Hyperglycemia and adverse pregnancy outcomes. N Engl J Med 2008;358(19):1991-2002.

105. Metzger B, Gabbe S, Persson B, Buchanan T, Catalano P, Damm P et al. International association of

diabetes and pregnancy study groups recommendations on the diagnosis and classification of hyperglycemia

in pregnancy. Diabetes Care 2010;33(3):676-82.

106. Chasan-Taber L, Marcus B, Stanek E, Ciccolo J, Marquez D, Solomon C et al. A randomized controlled

trial of prenatal physical activity to prevent gestational diabetes: design and methods. Journal of women's

health 2009;18(6):851-9.

107. Marín G, Marín BV. Research with Hispanic Populations. Newbury Park CA: Sage Publications; 1991.

108. Oropesa RS, Landale NS. Nonresponse in follow-back surveys of ethnic minority groups: an analysis of

the Puerto Rican Maternal and Infant Health Study. Matern Child Health J 2002;6(1):49-58.

109. American Diabetes Association. Gestational diabetes mellitus. Diabetes Care 2004;27 Suppl 1:S88-90.

110. Bandura A. *Self-Efficacy: The Exercise of Control*. New York: Freeman; 1997.

111. Prochaska JO, DiClemente CC. Stages and processes of self-change of smoking: toward an integrative

model of change. J Consult Clin Psychol 1983;51(3):390-5.

112. Rosal MC, Borg A, Bodenlos JS, Tellez T, Ockene IS. Awareness of diabetes risk factors and diabetes

prevention strategies among a sample of low-income Latinos with no known diagnosis of diabetes. The

Diabetes Educator [In Press].

113. Neighbors C, Marquez D, Marcus B. Leisure-time physical activity disparities among Hispanic

subgroups in the United States. Am J Public Health 2008;98(8):1460-4.

114. Committee to Reexamine IOM Pregnancy Weight Guidelines. Weight Gain During Pregnancy:

Reexamining the Guidelines. 2009.

115. ACOG Committee Obstetric Practice. ACOG Committee opinion. Number 267, January 2002: Exercise

during pregnancy and the postpartum period. Obstet Gynecol 2002;99(1):171-3.

116. Anonymous . American Diabetes Association position statement: evidence-based nutrition principles

and recommendations for the treatment and prevention of diabetes and related complications. Journal of the

American Dietetic Association 2002;102(1):109-118.

117. Fernandez S, Olendzki B, Rosal M. A dietary behaviors measure for use with low-income, Spanish speaking Caribbean Latinos with type 2 diabetes: the Latino Dietary Behaviors Questionnaire. J Am Diet Assoc 2011;111(4):589-99.

118. Tudor-Locke C, Lutes L. Why do pedometers work?: a reflection upon the factors related to

successfully increasing physical activity. Sports Medicine 2009;39(12):981-93.

119. Welk G, McClain J, Eisenmann J, Wickel E. Field validation of the MTI Actigraph and BodyMedia

armband monitor using the IDEEA monitor. Obesity 2007;15(4):918-28.

120. Fruin M, Rankin J. Validity of a multi-sensor armband in estimating rest and exercise energy

expenditure. Med Sci Sports Exerc 2004;36(6):1063-9.

121. Hustvedt B, Christophersen A, Johnsen L, Tomten H, McNeill G, Haggarty P et al. Description and

validation of the ActiReg: a novel instrument to measure physical activity and energy expenditure. Br J Nutr

2004;92(6):1001-8.

122. Jakicic J, Marcus M, Gallagher K, Randall C, Thomas E, Goss F et al. Evaluation of the SenseWear

Pro Armband to assess energy expenditure during exercise. Med Sci Sports Exerc 2004;36(5):897-904.

123. King G, Torres N, Potter C, Brooks T, Coleman K. Comparison of activity monitors to estimate energy

cost of treadmill exercise. Med Sci Sports Exerc 2004;36(7):1244-51.

124. St-Onge M, Mignault D, Allison D, Rabasa-Lhoret R. Evaluation of a portable device to measure daily

energy expenditure in free-living adults. Am J Clin Nutr 2007;85(3):742-9.

125. Berntsen S, Stafne S, Mrkved S. Physical activity monitor for recording energy expenditure in

pregnancy. Acta Obstet Gynecol Scand 2011 [epub ahead of print].

126. Chasan-Taber L, Schmidt MD, Roberts DE, Hosmer D, Markenson G, Freedson PS. Development and

validation of a Pregnancy Physical Activity Questionnaire. Med Sci Sports Exerc 2004;36(10):1750,6, ques

1757-60.

127. Nutrition Coordinating Center (NCC), University of Minnesota. Minnesota Nutrition Data System (NDS)

software. 1995;Food database version 10A; nutrient database version 25.

128. Marcus B, Lewis B, King T, Albrecht A, Hogan J, Bock B et al. Rationale, design, and baseline data for

Commit to Quit II: an evaluation of the efficacy of moderate-intensity physical activity as an aid to smoking

cessation in women. Prev Med 2003;36(4):479-92.

129. American College of Obstetricians and Gynecologists Committee on Practice Bulletins--Obstetrics.

ACOG Practice Bulletin. Clinical management guidelines for obstetrician-gynecologists. Number 30,

September 2001 (replaces Technical Bulletin Number 200, December 1994). Gestational diabetes. Obstet

Gynecol 2001;98(3):525-38.

130. King AC, Haskell WL, Taylor CB, Kraemer HC, DeBusk RF. Group- vs home-based exercise training in

healthy older men and women. A community-based clinical trial. JAMA 1991;266(11):1535-42.

131. Oldridge NB. Compliance and exercise in primary and secondary prevention of coronary heart disease:

a review. Prev Med 1982;11(1):56-70.

132. King AC, Blair SN, Bild DE, Dishman RK, Dubbert PM, Marcus BH et al. Determinants of physical

activity and interventions in adults. Med Sci Sports Exerc 1992;24(6 Suppl):S221-36.

133. Smith B, Cheung NW, Bauman A, Zehle K, McLean M. Postpartum physical activity and related

psychosocial factors among women with recent gestational diabetes mellitus. Diabetes Care

2005;28(11):2650-4.

134. Cardinal BJ, Sachs ML. Effects of mail-mediated, stage-matched exercise behavior change strategies

on female adults' leisure-time exercise behavior. J Sports Med Phys Fitness 1996;36(2):100-7.

135. Sacks DB. Carbohydrates. In: Burtis C, Ashwood E. Tietz Textbook of Clinical Chemistry. Third Edition

ed. Philadelphia, PA: Saunders; 1999. p. 750-808.

136. Rifai N, Cole TG, Iannotti E, Law T, Macke M, Miller R et al. Assessment of interlaboratory performance

in external proficiency testing programs with a direct HDL-cholesterol assay. Clin Chem 1998;44(7):1452-8.

137. Allain CC, Poon LS, Chan CS, Richmond W, Fu PC. Enzymatic determination of total serum

cholesterol. Clin Chem 1974;20(4):470-5.

138. Stinshoff K, Weisshaar D, Staehler F, Hesse D, Gruber W, Steier E. Relation between concentrations

of free glycerol and triglycerides in human sera. Clin Chem 1977;23(6):1029-32.

139. Sugiuchi H, Uji Y, Okabe H, Irie T, Uekama K, Kayahara N et al. Direct measurement of high-density

lipoprotein cholesterol in serum with polyethylene glycol-modified enzymes and sulfated alpha-cyclodextrin.

Clin Chem 1995;41(5):717-23.

140. Rifai N, Iannotti E, DeAngelis K, Law T. Analytical and clinical performance of a homogeneous

enzymatic LDL-cholesterol assay compared with the ultracentrifugation-dextran sulfate-Mg2 method. Clin

Chem 1998;44(6):1242-50.

141. Hasson R, Freedson P, Braun B. Postexercise insulin action in African-American women. J Natl Med

Assoc 2006;98(11):1832-9.

142. Metzger BE. Summary and recommendations of the Fifth International Workshop-Conference on

Gestational Diabetes Mellitus. Diabetes care 2007;30 Suppl 2:S251.

143. Chiang AN, Yang ML, Hung JH, Chou P, Shyn SK, Ng HT. Alterations of serum lipid levels and their

biological relevances during and after pregnancy. Life Sci 1995;56(26):2367-75.

144. Tropp LR, Erkut S, Coll CG, Alarcon O, Vazquez Garcia HA. Psychological Acculturation: Development

of a New Measure for Puerto Ricans on the U.S. Mainland. Educational and Psychological Measurement

1999;59(2):351-367.

145. Williams LM, Morrow B, Lansky A, Beck LF, Barfield W, Helms K et al. Surveillance for selected

maternal behaviors and experiences before, during, and after pregnancy. Pregnancy Risk Assessment

Monitoring System (PRAMS), 2000. MMWR Surveill Summ 2003;52(11):1-14.

146. Buysse DJ, Reynolds CF, Monk TH, Berman SR, Kupfer DJ. The Pittsburgh Sleep Quality Index: a new

instrument for psychiatric practice and research. Psychiatry Res 1989;28(2):193-213.

147. Cox JL, Holden JM, Sagovsky R. Detection of postnatal depression. Development of the 10-item

Edinburgh Postnatal Depression Scale. The British journal of psychiatry 1987;150:782-786.

148. Jadresic E, Araya R, Jara C. Validation of the Edinburgh Postnatal Depression Scale (EPDS) in

Chilean postpartum women. Journal of psychosomatic obstetrics and gynaecology 1995;16(4):187-191.

149. Stanek EJ3. Choosing a Pretest-Posttest Analysis. The American Statistician 1988;42(3):178,178-183.

150. Rosenbaum PR, Rubin DB. The central role of the propensity score in observational studies for causal

effects. Biometrika 1983;70:41-55.

151. Rubin DB. Basic ideas of multiple imputation for nonresponse. Survey Methodology 1986;12:37,37-47.

152. Little RJA, Rubin DB. Statistical Analysis with Missing Data. 2nd ed. New York: John Wiley and Sons;

2002.

153. Di Cianni G, Lencioni C, Volpe L, Ghio A, Cuccuru I, Pellegrini G et al. C-reactive protein and metabolic

syndrome in women with previous gestational diabetes. Diabetes Metab Res Rev 2007;23(2):135-40.

154. Retnakaran R, Qi Y, Connelly PW, Sermer M, Hanley AJ, Zinman B. Low adiponectin concentration

during pregnancy predicts postpartum insulin resistance, beta cell dysfunction and fasting glycaemia.

Diabetologia 2010;53(2):268-76.

155. Fahrenwald N, Atwood J, Walker S, Johnson D, Berg K. A randomized pilot test of "Moms on the

Move": a physical activity intervention for WIC mothers. Annals of behavioral medicine 2004;27(2):82-90.

156. Phelan S, Phipps M, Abrams B, Darroch F, Schaffner A, Wing R. Randomized trial of a behavioral

intervention to prevent excessive gestational weight gain: the Fit for Delivery Study. Am J Clin Nutr 2011;

Apr;93(4):772-9.

157. Cohen J. Statistical Power Analysis for the Behavioral Sciences. 2nd. ed. Hillsdale, NJ: Erlbaum; 1988.

158. van Hees V, Renstr?¶m F, Wright A, Gradmark A, Catt M, Chen K et al. Estimation of daily energy
expenditure in pregnant and non-pregnant women using a wrist-worn tri-axial accelerometer. PLoS ONE
2011;6(7):e22922-.
