# Supplementary material for: The impact of a randomized controlled trial of a lifestyle intervention on postpartum physical activity among at-risk hispanic women: Estudio PARTO
Source: PLoS One. 2020 Jul 24;15(7):e0236408. doi: 10.1371/journal.pone.0236408 (PMC7380594; doi:10.1371/journal.pone.0236408)
Supplement: Attachment — Submitted filename: renamed_f0cc7.docx [file pone.0236408.s003.docx]

**COMMENTS – Manuscript PONE-D-20-06832**

Title: “The Impact of a Randomized Controlled Trial of a Lifestyle Intervention on Postpartum Physical Activity among At-Risk Hispanic Women: Estudio PARTO”

**Important note: This review pertains only to ‘statistical aspects’ of the study and so** **‘****clinical aspects’ [like medical importance, relevance of the study,** **‘clinical significance and implication(s)’ of the whole study, etc.] are to be evaluated [should be assessed] separately/independently.**

This study is excellently planned & executed/conducted and manuscript is nicely drafted (no doubt about that). However, there are few suggestions [may please be considered as they may need to be clarified/told clearly at appropriate places in the text]:

1. In ‘Materials and methods - Study Design’ section it is said that “Randomization was stratified by the results of the diagnostic 100 g oral glucose tolerance test (OGTT)” but ‘what exactly is done’ is neither clear in further text nor from Figure 1 [Flow chart of participant recruitment and retention; Estudio PARTO 2013-2016]. It appears to be simple random allocation (may be by using permuted randomized blocks to ensure nearly same size in groups).
2. This study has taken data (it is a secondary data analysis then – please clarify) from some other trial {Estudio PARTO was a randomized controlled trial conducted in Western Massachusetts from 2013-17} for which the data were collected till 2017 then (my question is) ‘why this delay?’
3. Statistical comparison of baseline characteristics [last ‘p-value’ column in Table 1 (Distribution of Covariates according to Intervention Group; Estudio PARTO 2013-2016)] is not desirable at all [because even if P-value turns out to be significant (while comparing baseline characteristics despite random allocation), it is, by definition, a false positive] as you are supposed to be testing ‘randomization’ then, which in any single trial may not balance all baseline characteristics as ‘randomization’ is a sort of ‘insurance’ and not a guarantee scheme.

(Reference: Harrington D, D’Agostino RB, Gatsonis C, Hogan JW, Hunter DJ, Normand ST, et al. New guidelines for statistical reporting in the journal. N Engl J Med 2019;381:285-6)

4. It is known that ANCOVA has more ‘power’ than ‘change scores’ analyses [Reference: *‘The use of percentage change from baseline as an outcome in a controlled trial is statistically inefficient – a simulation study’* by Andrew Vickers in BMC Medical Research Methodology 2001, 1:6] displayed in table-3, however, it was necessary to use non-parametric tests [like Wilcoxon signed rank test for continuous variables, & McNemar test for categorical variables] on data yielded by tools [like Pregnancy Physical Activity Questionnaire (PPAQ) and the Self-Efficacy for Physical Activity Questionnaire]. Moreover, ANCOVA involves few assumptions which are many times not fulfilled (& are unlikely be fulfilled in this case).
5. Agreed that final analyses included Lifestyle Intervention (N=71) & Health & Wellness Intervention (N=79) but baseline data in table-1 should have all who were randomized to start with [were randomized to a Lifestyle Intervention (LI, n=100) or to a comparison Health and Wellness (HW, n=104) group]. Is not that so? ‘Covariate assessment’ section states that “Demographic and behavioral characteristics were collected at baseline via standardized questionnaires”. ‘Statistical analyses’ section states that “We used an intent-to-treat analysis to evaluate the impact of the LI on the adoption and maintenance of increased PA and self-efficacy as compared to the HW group”.

Otherwise everything else [including Using Mixed Models] is faultless. Except above minor ‘revision’ points, I recommend the publication of this article in our journal.
