# Supplementary material for: The impact of a randomized controlled trial of a lifestyle intervention on postpartum physical activity among at-risk hispanic women: Estudio PARTO
Source: PLoS One. 2020 Jul 24;15(7):e0236408. doi: 10.1371/journal.pone.0236408 (PMC7380594; doi:10.1371/journal.pone.0236408)
Supplement: Attachment — Submitted filename: Response to reviewer comments.docx [file pone.0236408.s004.docx]

**Manuscript PONE-D-20-06832**

**Editor’s Comments**

1. **Specifically, the reviewers suggests some changes in the statistical methods section to clarify some steps taking in analysing the data.**

We have revised the statistical methods section to clarify steps taken in analyzing the data as described in the response to reviewer comments below.

2. **Please ensure that your manuscript meets PLOS ONE's style requirements, including those for file naming.**

We confirm that our manuscript meets PLOS ONE's style requirements including those for file naming.

3. **We noticed you have some minor occurrence of overlapping text with the following previous publication(s), which needs to be addressed:**

[**https://www.sciencedirect.com/science/article/pii/S1521693414001618?via%3Dihub**](https://www.sciencedirect.com/science/article/pii/S1521693414001618?via%3Dihub)
[**https://bmcpregnancychildbirth.biomedcentral.com/articles/10.1186/1471-2393-14-100**](https://bmcpregnancychildbirth.biomedcentral.com/articles/10.1186/1471-2393-14-100)
[**https://bmchealthservres.biomedcentral.com/articles/10.1186/s12913-019-4207-x**](https://bmchealthservres.biomedcentral.com/articles/10.1186/s12913-019-4207-x)

**In your revision ensure you cite all your sources (including your own works), and quote or rephrase any duplicated text outside the methods section. Further consideration is dependent on these concerns being addressed.**

We have revised the manuscript to rephrase any duplicated text outside the methods section and now cite our sources, including our own work.

4. **Please include captions for your Supporting Information files at the end of your manuscript, and update any in-text citations to match accordingly. Please see our Supporting Information guidelines for more information:** [**http://journals.plos.org/plosone/s/supporting-information**](about:blank)

We now include captions for our supporting information files at the end of the manuscript.

**Review Comments to the Author**

**Reviewer #1: Important note: This review pertains only to ‘statistical aspects’ of the study and so** **‘****clinical aspects’ [like medical importance, relevance of the study,** **‘clinical significance and implication(s)’ of the whole study, etc.] are to be evaluated [should be assessed] separately/independently. This study is excellently planned & executed/conducted and manuscript is nicely drafted (no doubt about that). However, there are few suggestions [may please be considered as they may need to be clarified/told clearly at appropriate places in the text]**

We thank the reviewer for this comment and address their suggestions below.

1. **In ‘Materials and methods - Study Design’ section it is said that “Randomization was stratified by the results of the diagnostic 100 g oral glucose tolerance test (OGTT)” but ‘what exactly is done’ is neither clear in further text nor from Figure 1 [Flow chart of participant recruitment and retention; Estudio PARTO 2013-2016]. It appears to be simple random allocation (may be by using permuted randomized blocks to ensure nearly same size in groups).**

We have revised the Study Design section (page 6, paragraph 1) to now clarify that randomization was stratified by the results of the diagnostic 100 g oral glucose tolerance test (OGTT) using thresholds defined by the American Diabetes Association (ADA): 1) no glucose values meeting or exceeding the ADA thresholds; or 2) one or more glucose values meeting or exceeding the ADA thresholds) [24].

2. **This study has taken data (it is a secondary data analysis then – please clarify) from some other trial {Estudio PARTO was a randomized controlled trial conducted in Western Massachusetts from 2013-17} for which the data were collected till 2017 then (my question is) ‘why this delay?’**

We apologize for our lack of clarity. We have revised the Study Design section (page 5, paragraph 2) to now clarify that this is a primary data analysis. In other words, one of the primary aims of Estudio PARTO was to evaluate the impact of the intervention on PA and self-efficacy. The reviewer is correct that data was collected on active participants until the end of 2017. We then received a no-cost extension from NIH to allow us additional time to conduct data analysis and interpretation. This, combined with the availability of the coauthors, led to our finalizing the manuscript at the end of 2019 and submitting it in early 2020.

3. **Statistical comparison of baseline characteristics [last ‘p-value’ column in Table 1 (Distribution of Covariates according to Intervention Group; Estudio PARTO 2013-2016)] is not desirable at all [because even if P-value turns out to be significant (while comparing baseline characteristics despite random allocation), it is, by definition, a false positive] as you are supposed to be testing ‘randomization’ then, which in any single trial may not balance all baseline characteristics as ‘randomization’ is a sort of ‘insurance’ and not a guarantee scheme.**

**(Reference: Harrington D, D’Agostino RB, Gatsonis C, Hogan JW, Hunter DJ, Normand ST, et al. New guidelines for statistical reporting in the journal. N Engl J Med 2019;381:285-6)**

The reviewer makes an excellent point and we have revised Table 1 to no longer include the p-value column.

4. **It is known that ANCOVA has more ‘power’ than ‘change scores’ analyses [Reference: *‘The use of percentage change from baseline as an outcome in a controlled trial is statistically inefficient – a simulation study’* by Andrew Vickers in BMC Medical Research Methodology 2001, 1:6] displayed in table-3, however, it was necessary to use non-parametric tests [like Wilcoxon signed rank test for continuous variables, & McNemar test for categorical variables] on data yielded by tools [like Pregnancy Physical Activity Questionnaire (PPAQ) and the Self-Efficacy for Physical Activity Questionnaire]. Moreover, ANCOVA involves few assumptions which are many times not fulfilled (& are unlikely be fulfilled in this case).**

We agree with the reviewer regarding their approval of our change scores analysis and our use of non-parametric tests.

5. **Agreed that final analyses included Lifestyle Intervention (N=71) & Health & Wellness Intervention (N=79) but baseline data in table-1 should have all who were randomized to start with [were randomized to a Lifestyle Intervention (LI, n=100) or to a comparison Health and Wellness (HW, n=104) group]. Is not that so? ‘Covariate assessment’ section states that “Demographic and behavioral characteristics were collected at baseline via standardized questionnaires”. ‘Statistical analyses’ section states that “We used an intent-to-treat analysis to evaluate the impact of the LI on the adoption and maintenance of increased PA and self-efficacy as compared to the HW group”.**

We have revised Table 1 to now include all the participants who were randomized to start with (LI, n=100) and (HW, n=104) as per the reviewer’s suggestion.

6. **Otherwise everything else [including Using Mixed Models] is faultless. Except above minor ‘revision’ points, I recommend the publication of this article in our journal.**

We thank the reviewer for this comment.

**Reviewer #2: Summary:
This study assess the change in physical activity (PA) of a RCT using an individually-tailored approach for postpartum Hispanic women. The study design and statistics are exceptional. This study is an important addition to the literature because it focuses on Hispanic, postpartum women at high risk for chronic disease and assess the use of a targeted intervention, which is more culturally appropriate and has the potential to advance the field of PA. I applaud these authors for this work and hope to review more studies like this in the future.**

We thank the reviewer for this comment.

**Minor Revisions:**

1. **Thank you for including your protocol in the additional information. Please include in the text more information on the specific intervention components that differed between the LI and HW groups. You state that your attention control group received the same number of sessions, calls, and mailers – this emphasized the need to know as much information about the difference in interventions as possible.**

We have revised the Methods section to now include more detail about Health and Wellness intervention received by the attention control group (page 8, paragraph 3).

2. **State future directions for this work, including potential public health interventions that should be considered by practitioners.**

We have revised the Discussion section to now describe future directions for this work including potential future interventions (page 23, paragraph 2).
